# Sigscores: summary scores for molecular signatures in R

**DOI:** 10.1093/bioadv/vbag021

**Published:** 2026-01-22

**Authors:** Alessandro Barberis, Francesca M Buffa

**Affiliations:** Computational Biology and Integrative Genomics Lab, Department of Oncology, University of Oxford, Oxford, OX3 7DQ, United Kingdom; IGF Lab, Nuffield Department of Surgical Sciences, University of Oxford, Oxford, OX3 9DU, United Kingdom; Computational Biology and Integrative Genomics Lab, Department of Oncology, University of Oxford, Oxford, OX3 7DQ, United Kingdom; Department of Computing Sciences and Bocconi Institute for Data Science and Analytics, Bocconi University, Milan, 20136, Italy; AI and Systems Biology Lab, IFOM—Istituto Fondazione di Oncologia Molecolare ETS, Milan, 20139, Italy

## Abstract

**Summary:**

The rapid expansion of multi-omics data has enabled the development of molecular signatures—coordinated patterns of molecular features that serve as powerful biomarkers for diagnosis, prognosis, and therapeutic decision-making. Despite their potential, many published signatures suffer from limited reproducibility and narrow applicability, partly due to challenges in summarizing complex, multi-feature profiles into a single, statistically sound and biologically meaningful score. Here, we introduce sigscores, an R package that streamlines the computation of summary scores for molecular signatures. Building on the quality control principles of our earlier tool, sigQC, sigscores supports an extensive array of scoring metrics—including measures of central tendency, dispersion, and aggregation. It incorporates a resampling framework to generate empirical null distributions for rigorous significance assessment and provides integrated visualization tools for diagnostic evaluation. Optimized for parallel execution on multi-core systems, sigscores is well-suited for both exploratory research and high-throughput large-scale applications.

**Availability and implementation:**

Source code freely available for download on GitHub at https://github.com/alebarberis/sigscores, implemented in R and supported on MacOS and MS Windows.

## 1 Introduction

Technological advances and steep decline in high-throughput sequencing costs have led to a surge in biomedical data production at different resolution, allowing unprecedented views of biological systems at molecular level. Initially driven by genomics, followed by transcriptomics, research has now expanded into a multi-omics paradigm, where multiple omics, such as transcriptomics, proteomics, and metabolomics, are often incorporated into routine biological investigations (Hasin *et al.* 2017). These developments have paved the way for the identification of molecular signatures, also referred to in the literature as gene signatures or gene sets, broadly defined here as sets of molecular features (e.g. mRNA transcripts, proteins, metabolites, genetic variants) exhibiting clear coordinated patterns representative of particular biological phenotypes, states, processes, or outcomes. In recent years, the use of gene signatures has greatly expanded, revolutionizing our understanding of development, pathogenesis, and disease, and in some cases impacting clinical practice. For example, gene signatures are now commonly applied in fields such as oncology and immunology for disease diagnosis, prognosis, treatment selection, and the identification of potential therapeutic targets. Their significance has grown even further with the advent of technologies like single-cell RNA sequencing and spatial transcriptomics, which enable the identification of distinct cell populations, their biological functions, and their interactions. However, despite some exceptions, many of the reported signatures suffer from narrow applicability, poor reproducibility across independent datasets, and insufficient sensitivity and specificity for clinical deployment (Michiels *et al.* 2005, Venet *et al.* 2011).

To streamline the evaluation of statistical properties of gene signatures across unrelated datasets and to facilitate a better understanding of why certain signatures may fail to meet biological and/or clinical requirements, we previously developed sigQC (Dhawan *et al.* 2019). This R package was designed as a robust quality control framework, providing researchers with valuable diagnostic tools to assess the reliability of gene signatures before they are used in downstream analyses.

Another central problem that limits the applicability of gene signatures in the clinical setting is the difficulty of summarizing these complex multi-features profiles into single scores that are not only interpretable but also actionable. While we introduced six summarization scores in sigQC for simplicity and usability, the list was not exhaustive as several other methods have been proposed in the literature. Furthermore, there is no standardization in the application of these methods, nor guidelines on how to apply and assess them. To address this, we introduce sigscores (https://github.com/alebarberis/sigscores/), an R package designed to summarize gene signatures into single, interpretable scores while providing visualization capabilities and resampling-based significance assessment. Building on the design principles of sigQC, sigscores offers a flexible, robust framework that supports the computation of a broad range of scoring metrics, custom data transformations, and implements resampling-based assessments using random data or signatures. The package is implemented in R and has been designed to run efficiently on multi-core systems, making it suitable for both exploratory research and high-throughput, large-scale statistical and clinical studies.

## 2 Design and implementation

sigscores streamlines the conversion of complex gene signatures into single scores by integrating data transformation, score computation, and statistical evaluation into one cohesive workflow. The function computeSigScores() provides easy access to the computation of all the available summary metrics, allowing the user to select parallel or serial execution on a multi-core machine. The scores are computed by using Scorer functions having a consistent interface to facilitate usage. Each Scorer accepts a data transformation function in input, which can be used to transform the data before the computation of the scores. computeSigScores() also facilitates the assessment of the robustness and significance of the scores by allowing the computation of the scores on resampled data and/or by using random signatures.

### 2.1 Data transformation and scores

The software supports an extensive range of scoring methods. In addition to the six metrics inherited from sigQC (i.e. arithmetic mean, median, first principal component, gene set variation analysis, single-sample gene set enrichment analysis, pathway-level analysis of gene expression), sigscores includes additional measures of total quantity, central tendency, and data variability, such as sum, weighted sum, trimmed mean, weighted mean, mode, midrange, midhinge, trimean, interquartile range, interquartile mean, median absolute deviation, and average absolute deviation. Moreover, we also started including scores retrieved from the literature, such as the one based on z-standardized values as computed by Lee *et al.* (2008). Each Scorer function is designed with a unified interface and accepts a data transformer function as an argument, facilitating the automated pre-processing of data prior to score computations. This approach is particularly useful in scenarios where—starting from the same dataset—different transformations need to be applied before computing multiple summary scores. It not only automates the process but also allows users to define custom signature scores by combining data transformation and summarization, thereby enhancing computational possibilities. Currently, sigscores provides three built-in data transformation functions: a step function, a quantile normalization function, and a z-standardization function.

### 2.2 Sampling and random signatures

A critical aspect of interpreting gene signature scores is determining whether the observed values reflect coordinated biological signal or arise from random variation. To address this, sigscores implements resampling-based approaches that provide non-parametric, model-agnostic assessments of score robustness and deviation from random expectation. Such approaches are widely used in genomics and gene set analysis, where distributional assumptions (e.g. normality or independence) are often violated and analytical null models are unavailable. Two complementary resampling strategies are supported. First, simulated data sampling perturbs the observed data by resampling molecular feature values with or without replacement, enabling evaluation of score stability under data variability. Second, random feature selection, where sets of features matching the size of the original signature are drawn from a background pool, allowing assessment of whether an observed score exceeds what would be expected for a random set of features. Both strategies lead to the computation of random summary scores that can be aggregated to form empirical null distributions, against which the observed score can be compared. The built-in function computeSignificance() facilitates the calculation of inferential quantities such as achieved significance level (ASL), standard errors, and confidence intervals, following established bootstrap and resampling methodologies. As the appropriate inferential framework and multiple testing correction depend on the downstream analytical context—such as the number of signatures or scoring methods evaluated, study design, and intended use of the scores—our package focuses on generating empirical null distributions and diagnostic summaries, while leaving final hypothesis testing decisions and adjustment strategies to the user. By facilitating the non-parametric evaluation of individual score reliability, the sampling functionality of sigscores is a valuable asset for researchers looking to make data-driven conclusions.

### 2.3 Integrated visualization

**Figure 1:**
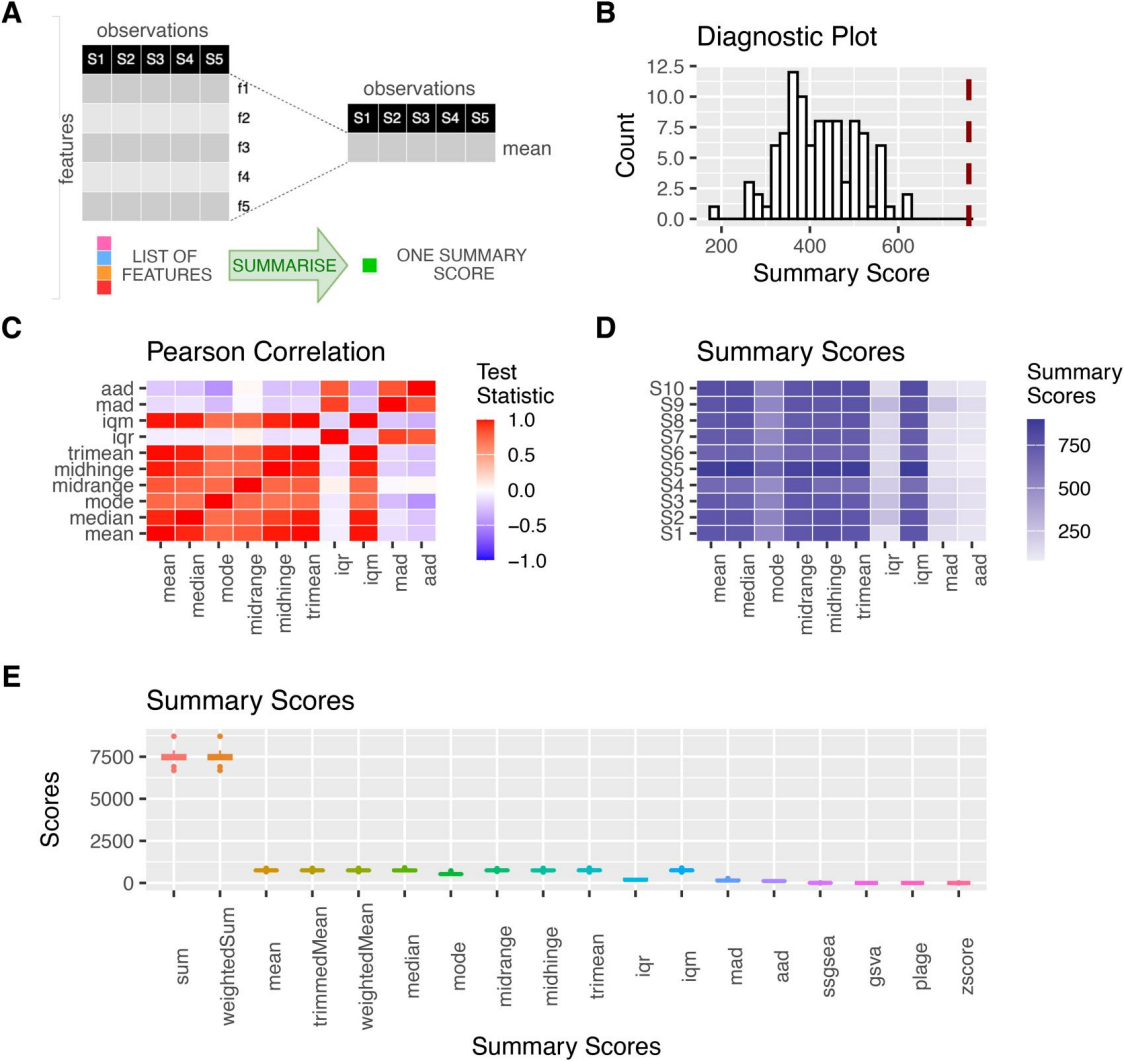
Comprehensive visual summary of sigscores' functionality and output. (A) sigscores facilitates the summarization of gene signatures into single scores. (B) An empirical null distribution of random signatures, displayed as a histogram, with the true score overlaid as a vertical line to highlight its statistical significance. (C) A heatmap depicting correlations between different scoring metrics, providing insight into their relationships and the robustness of the scoring methods. A high degree of correlation suggests that a given signature is well-suited for summarization. (D) A heatmap of computed signature scores across samples, enabling the identification of patterns, similarities, and differences between conditions. (E) Boxplots showing the distribution of computed scores across samples, facilitating the comparison of scoring methods.

To facilitate the inspection of results and the comparison of scores across samples, sigscores provides a suite of plotting functions that generate publication-quality figures (Figure 1). Built on widely used visualization packages such as ComplexHeatmap (Gu *et al.* 2016) and ggplot2 (Wickham 2016), the available graphical outputs include (i) boxplots and scatter plots, for comparing score distributions, (ii) heatmaps, for visually inspecting the expression patterns underlying gene signatures, and (iii) diagnostic plots, illustrating the empirical null distributions derived from resampling, thereby providing a visual benchmark for the observed scores. By integrating these visualization tools seamlessly into its workflow, sigscores ensures that users can effectively interpret and communicate their results.

## 3 Discussion

Expanding our initial work on sigQC, sigscores represents a significant advance in the computational analysis of gene signatures by providing a unified framework for summarizing complex feature profiles into single, interpretable scores. Its primary aim is to support the computation, systematic evaluation, comparison, and interpretation of summary scores by assessing their robustness to data perturbation, consistency across alternative scoring methods, and deviation from random expectation using resampling-based null models. These diagnostics are intended to guide the selection of appropriate signature-score combinations and to highlight potential limitations before scores are incorporated into downstream analyses, such as phenotype association studies, predictive models, or clinical decision-making workflows. Scores identified as robust, consistent, and significantly different from random expectation can be prioritized for further use, whereas potential limitations highlighted by the diagnostics may inform adjustments or suggest further investigation. By addressing both computational and statistical challenges, the package is ideally suited for applications ranging from basic research to clinical biomarker evaluation.

One of the primary advantages of sigscores is its modular design, which facilitates the use of a diverse range of scoring methods while ensuring that the overall workflow remains consistent and user-friendly. The ability to integrate custom data transformation functions ensures that the input data can be optimally pre-processed, also enhancing the possibility of computed scores. In addition to its flexible scoring capabilities, sigscores is distinguished by its built-in statistical evaluation framework. The generation of empirical null distributions via resampling and random feature selection provides a rigorous method for assessing the significance of observed scores, bolstering the statistical confidence in the results and also facilitating comparative analyses. The integrated visualization tools further enhance the utility of sigscores by enabling researchers to create clear, publication-quality figures directly from their analysis workflow. Another noteworthy aspect of sigscores is its support for both parallel and serial execution. By combining computation, statistical evaluation, and visualization within a single package, sigscores significantly streamlines the process of gene signature analysis, reducing the need for learning multiple disjointed tools and manual data handling.

To illustrate sigscores’ practical utility, we showcase here its application in a recent study (Di Giovannantonio *et al.* 2025) where we assessed 70 hypoxia gene expression signatures across 104 cell lines and over 5000 clinical tumor samples spanning 10 solid tumor types. This comprehensive analysis aimed to determine the most effective methods for summarizing hypoxia signatures into single, interpretable scores, facilitating the inference of hypoxic phenotypes in various biological and clinical contexts. Utilizing sigscores, we evaluated 14 different scoring methods for each signature and found that both signature selection and scoring methodology significantly influenced hypoxia prediction. Notably, the Buffa *et al.* (2010) signature combined with the mean scoring method and the Ragnum *et al.* (2015) signature with the interquartile mean demonstrated superior performance in distinguishing hypoxic from normoxic conditions in tumor samples. These findings underscore the importance of selecting appropriate signature-score combinations for accurate biological inference and highlight the utility of sigscores in optimizing biomarker evaluation and clinical application.

Looking forward, several avenues exist for further development of sigscores. Future iterations may include support for additional scoring functions or the integration of machine learning techniques to capture complex relationships. Moreover, expanding the range of available visualization tools to include interactive web-based dashboards could further broaden the applicability of the package, particularly for collaborative projects or clinical settings where real-time data exploration is valuable.

## 4 Conclusion

In conclusion, sigscores is a powerful and flexible tool that addresses a critical need in the field of computational biology. By providing a unified framework for the computation, evaluation, and visualization of gene signature scores, sigscores not only simplifies the analytical workflow but also enhances the reliability and interpretability of the results. The package is freely available on GitHub, and detailed installation instructions and a comprehensive Getting Started guide can be found at https://alebarberis.github.io/sigscores/index.html. We believe that sigscores will serve as a valuable resource for both basic research and clinical applications, contributing to the ongoing efforts to translate genomic insights into tangible health benefits.

## Data Availability

No new data were generated or analysed in support of this research.
